# Prioritizing Health-Sector Interventions for Noncommunicable Diseases and Injuries in Low- and Lower-Middle Income Countries: National NCDI Poverty Commissions

**DOI:** 10.9745/GHSP-D-21-00035

**Published:** 2021-09-30

**Authors:** Neil Gupta, Ana Mocumbi, Said H. Arwal, Yogesh Jain, Abraham M. Haileamlak, Solomon T. Memirie, Nancy C. Larco, Gene F. Kwan, Mary Amuyunzu-Nyamongo, Gladwell Gathecha, Fred Amegashie, Vincent Rakotoarison, Jones Masiye, Emily Wroe, Bhagawan Koirala, Biraj Karmacharya, Jeanine Condo, Jean Pierre Nyemazi, Santigie Sesay, Sarah Maogenzi, Mary Mayige, Gerald Mutungi, Isaac Ssinabulya, Ann R. Akiteng, Justice Mudavanhu, Sharon Kapambwe, David Watkins, Ole Norheim, Julie Makani, Gene Bukhman, NCDI Poverty National Commissions Authorship Group; NCDI Poverty Network Secretariat

**Affiliations:** a Partners In Health NCD Synergies, Boston, MA, USA.; b Division of Global Health Equity, Brigham & Women’s Hospital, Boston MA, USA.; c Program in Global NCDs and Social Change, Department of Global Health and Social Medicine, Harvard Medical School, Boston, MA, USA.; d Universidade Eduardo Mondlane, Maputo, Mozambique; Instituto Nacional de Saúde, Maputo, Mozambique.; e Afghan Ministry of Public Health, Kabul, Afghanistan.; f Sangwari, Surguja, Chhattisgarh, India.; g Ethiopia Ministry of Health, Addis Ababa, Ethiopia.; h Addis Center for Ethics and Priority Setting, Addis Ababa, Ethiopia.; i Fondation Haïtienne de Diabète et de Maladies Cardio-Vasculaires, Port-au-Prince, Haiti.; j Section of Cardiovascular Medicine, Boston University School of Medicine, Boston, MA, USA.; k African Institute for Health and Development, Nairobi, Kenya.; l Kenya Ministry of Health, Nairobi, Kenya.; m Liberia Ministry of Health, Monrovia, Liberia.; n Madagascar Ministère de la Santé Publique, Antananarivo, Madagascar.; o Malawi Ministry of Health, Lilongwe, Malawi.; p Manmohan Cardiothoracic Vascular and Transplant Center Institute of Medicine, Kathmandu, Nepal.; q Department of Public Health and Community Programs, Kathmandu University School of Medical Sciences, Dhulikhel, Nepal.; r School of Public Health, University of Rwanda, Kigali, Rwanda.; s World Health Organization, Geneva, Switzerland.; t Sierra Leone Ministry of Health and Sanitation, Freetown, Sierra Leone.; u Tanzania Ministry of Health, Community Development, Gender, Elderly and Children, Dodoma, Tanzania.; v National Institute for Medical Research, Dar es Salaam, Tanzania.; w Uganda Ministry of Health, Kampala, Uganda.; x Uganda Initiative for Integrated Management of Non-Communicable Diseases, Kampala, Uganda.; y Makerere University College of Health Sciences, Kampala, Uganda.; z Zimbabwe Ministry of Health & Child Care, Harare, Zimbabwe.; aa Zambia Ministry of Health, Lusaka, Zambia.; bb Division of General Internal Medicine, Department of Medicine and Department of Global Health, University of Washington, Seattle, WA, USA.; cc Department of Global Public Health and Primary Care, University of Bergen, Bergen, Norway.; dd Department of Global Health and Population, Harvard T H Chan School of Public Health, Harvard University, Boston, MA, USA.; ee Muhimbili University of Health and Allied Sciences, Dar es Salaam, Tanzania.; ff Division of Cardiovascular Medicine, Department of Medicine, Brigham & Women’s Hospital, Boston, MA, USA.

## Abstract

Health sector priorities and interventions to prevent and manage noncommunicable diseases and injuries (NCDIs) in low- and lower-middle-income countries (LLMICs) have primarily adopted elements of the World Health Organization Global Action Plan for NCDs 2013–2020. However, there have been limited efforts in LLMICs to prioritize among conditions and health-sector interventions for NCDIs based on local epidemiology and contextually relevant risk factors or that incorporate the equitable distribution of health outcomes. The *Lancet* Commission on Reframing Noncommunicable Diseases and Injuries for the Poorest Billion supported national NCDI Poverty Commissions to define local NCDI epidemiology, determine an expanded set of priority NCDI conditions, and recommend cost-effective, equitable health-sector interventions. Fifteen national commissions and 1 state-level commission were established from 2016–2019. Six commissions completed the prioritization exercise and selected an average of 25 NCDI conditions; 15 conditions were selected by all commissions, including asthma, breast cancer, cervical cancer, diabetes mellitus type 1 and 2, epilepsy, hypertensive heart disease, intracerebral hemorrhage, ischemic heart disease, ischemic stroke, major depressive disorder, motor vehicle road injuries, rheumatic heart disease, sickle cell disorders, and subarachnoid hemorrhage. The commissions prioritized an average of 35 health-sector interventions based on cost-effectiveness, financial risk protection, and equity-enhancing rankings. The prioritized interventions were estimated to cost an additional US$4.70–US$13.70 per capita or approximately 9.7%–35.6% of current total health expenditure (0.6%–4.0% of current gross domestic product). Semistructured surveys and qualitative interviews of commission representatives demonstrated positive outcomes in several thematic areas, including understanding NCDIs of poverty, informing national planning and implementation of NCDI health-sector interventions, and improving governance and coordination for NCDIs. Overall, national NCDI Poverty Commissions provided a platform for evidence-based, locally driven determination of priorities within NCDIs.

## Abstract (precis)

Noncommunicable Disease and Injury (NCDI) Poverty Commissions in 16 low- and middle-income countries provided evidence-based recommendations on a local, expanded set of priority NCDIs and health-sector interventions needed in national initiatives to attain universal health coverage. These commissions provide a collective platform for policy, research, and advocacy efforts to improve coverage of cost-effective and equitable health-sector interventions for populations living in extreme poverty.

## Teaser

**Key Findings:**

- National Noncommunicable Disease and Injury (NCDI) Poverty Commissions were established in 16 low- and lower-middle-income countries (LLMICs) to define local NCDI epidemiology, determine an expanded set of priority NCDI conditions, and recommend cost-effective, equitable health-sector interventions.
- Commissions prioritized an average of 35 health-sector interventions estimated to cost an additional US$4.70–US$13.70 per capita or approximately 9.7%–35.6% of current total health expenditure (0.6%–4.0% of current gross domestic product).
- Commissions demonstrated positive outcomes in understanding NCDIs of poverty, informing national planning and implementation of NCDI health-sector interventions, and improving governance and coordination for NCDIs.

**Key Implications:**

- Policy makers should use findings from the National NCDI Poverty Commissions to provide evidence-based, locally driven determination of priorities to guide health-sector policies, programs, and investments for NCDIs.
- External financing will be required to bridge the gap in financing for NCDIs to achieve universal health coverage in LLMICs.

## BACKGROUND

Noncommunicable diseases and injuries (NCDIs) are a major contributor to morbidity and mortality in low- and lower-middle-income countries (LLMICs).^1^ The World Health Organization (WHO) Global Action Plan for Prevention and Control of Noncommunicable Diseases (NCDs) 2013–2020 emphasizes 4 behavioral risk factors (tobacco use, unhealthy diets, physical inactivity, and harmful use of alcohol) in the context of 4 disease groups (cardiovascular diseases, diabetes, cancer, and chronic respiratory diseases), subsequently expanded to include air pollution and mental health disorders.^2^^,^^3^ However, there is increasing evidence that this framework does not adequately represent the diverse and comprehensive set of risk factors and NCDIs comprising the disease burden in LLMICs.^4^^,^^5^ The conditions comprising this burden in resource-constrained settings are diverse, and infectious diseases and conditions related to poverty comprise a large component of associated risk factors.^6^^,^^7^ These conditions not only result in a large burden of disease in LLMICs, but due to younger population demographics, delays to diagnosis, and limited service availability, they tend to occur earlier and more severely in these populations.^8^ Prolonged chronicity of these conditions along with dependency on out-of-pocket payments for NCDI services result in dramatic impoverishment and productivity losses as compared to other disease areas.^9^^,^^10^

To date, NCDI strategic plans and frameworks in LLMICs have been largely influenced by elements of the existing global action plan and monitoring framework.^11^ Efforts to adapt or contextualize the WHO Global Action Plan for Prevention and Control of NCDs to the national burden of NCDI conditions or health system capacities in LLMICs have been inadequately prioritized or resourced.^11^^–^^13^ Interventions proposed to avert the burden of NCDIs have focused on primary and secondary prevention of conditions due to behaviorally mediated risk factors and have not frequently considered a broader range of NCDIs and their associated risk factors.^11^ Furthermore, these interventions have been largely evaluated and selected based on measures of cost-effectiveness and feasibility within resource-constrained environments and have not traditionally included measurements of equitable distribution of health outcomes at country-level.^14^

Efforts to adapt or contextualize the WHO Global Action Plan for Prevention and Control of NCDs to the national burden of NCDI conditions or health system capacities in LLMICs have been inadequately prioritized or resourced.

The *Lancet* Commission on Reframing Noncommunicable Diseases and Injuries for the Poorest Billion (“*Lancet* NCDI Poverty Commission”) was established to assess the nature of the NCDI burden among the poorest billion people in the world, assure that sustainable financing is not a bottleneck to NCDI prevention and treatment among the world’s poorest, and expand the NCD movement and the global health agenda to urgently address NCDIs among the poorest billion.^5^ The *Lancet* NCDI Poverty Commission additionally aimed to work with stakeholders in a diverse group of countries to develop actionable pro-poor pathways for expansion of NCDIs interventions. To achieve these objectives, the *Lancet* NCDI Poverty Commission established and supported national and subnational NCDI Poverty Commissions in countries with high rates of extreme poverty.

In this article, we first describe the structure and analytic framework used by the national NCDI Poverty Commissions (termed “commissions”). We then provide a synthesis of the characteristics, findings, and recommendations reported by the commissions. Lastly, we report results of a semistructured survey and qualitative interviews conducted with key informants from the commissions regarding short-term outcomes of the commissions in 11 thematic areas corresponding to major objectives of the *Lancet* NCDI Poverty Commission framework.

## METHODS

### Commission Establishment and Capacity Building

The *Lancet* NCDI Poverty Commission was established in 2015 and supported administratively and technically by a central secretariat at the Program in Global NCDs and Social Change at Harvard Medical School. The measure used by the *Lancet* NCDI Poverty Commission to assess poverty was a global multidimensional poverty index based on indicators of living standards and education obtained through routine and standardized household surveys.^15^^,^^16^ The “poorest billion” were defined as households with deprivations in at least 5 of the 8 indicators included in this modified index.

Key individuals active in clinical, research, programmatic, or governance aspects of the NCDI health sector response from a diverse representation of this group of countries were invited to review the analytic framework developed for the *Lancet* NCDI Poverty Commission and propose adaptations to reflect national objectives (detailed in Analytic Approach). The *Lancet* NCDI Poverty Commission then invited the Ministry of Health of each key informant’s respective country to formally nominate and commission a national group of multisectoral experts in NCDIs to undertake an analytic and consultative process with technical, financial, and administrative support from the *Lancet* NCDI Poverty Commission secretariat.

From January to December 2016, 10 countries were invited to apply for support to establish an NCDI Poverty Commission (Round I). Five additional countries and 1 subnational region (Chhattisgarh State, India) were invited to establish commissions from October 2018 to September 2019 (Round II). Each Ministry of Health nominated 1 to 2 chairpersons to lead the commission, a coordinator to administer commission activities and communications, and approximately 20–30 multisectoral experts in the area of NCDIs, including policy makers, clinicians, researchers, patient group advocates, health economists, and health-sector planning experts.

All national commissioners were invited to participate in a series of 6 online “Knowledge Exchange” teleconferences jointly organized by the *Lancet* NCDI Poverty Commission and the World Bank. These teleconferences consisted of didactic presentations from global experts and participatory discussions facilitated by commission leads.^17^ Subregional meetings in East Africa (Kigali, Rwanda, March 2018) and Southern Africa (Maputo, Mozambique, June 2018) and a 4-day workshop entitled “The NCDI Poverty Commission Initiators’ Workshop” (Dubai, United Arab Emirates, 2018) were held to foster collaboration and knowledge sharing among commissions.^18^

### Analytic Approach by Commissions

**FIGURE 1:**
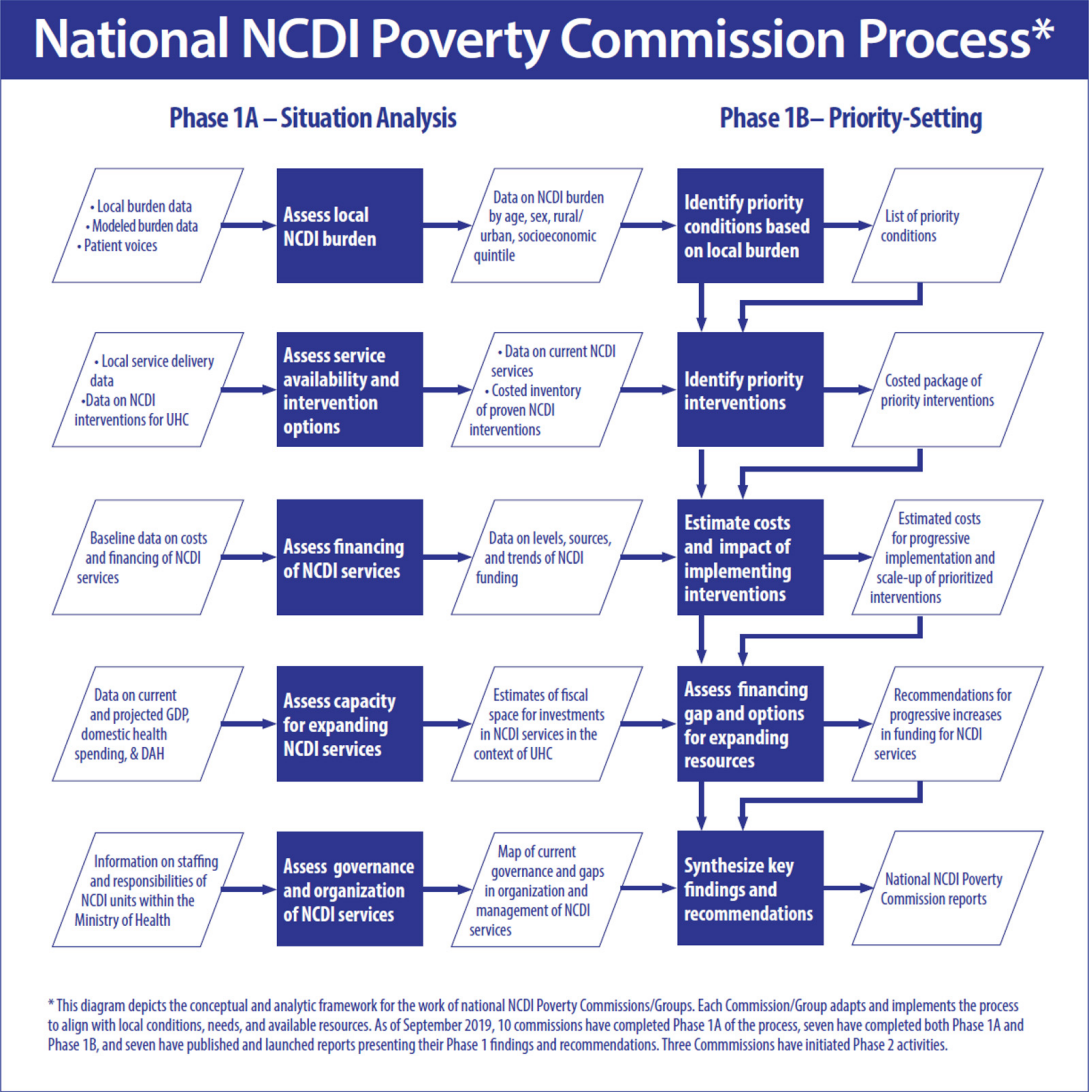
National Noncommunicable Diseases and Injuries Poverty Commission Analytic Framework Abbreviations: DAH; developmental assistance for health; GDP, gross domestic product; NCDI, noncommunicable diseases and injuries; UHC, universal health coverage.

An analytic framework to achieve national commission objectives was developed (Figure 1). Phase 1A focused on aggregating a range of available data related to the burden of disease, service availability, financing, and governance of NCDI services in each country (Supplement Table 1). Descriptive statistics regarding population demographics were obtained from the Global Burden of Disease study (GBD) 2017 and World Bank World Development Indicators.^1^^,^^19^ Data regarding health expenditures were obtained from national health accounts compiled and available from the WHO Global Health Expenditure Database and human resources from the WHO Global Health Workforce Statistics.^20^^,^^21^

In Phase 1B, national commissions convened a set of deliberations and meetings to review the available evidence in NCDIs and make recommendations for local policy. This prioritization exercise was structured using the principles of priority setting established by the WHO Consultative Group on Equity and Universal Health Coverage.^22^ To develop a list of priority NCDI conditions, the commissions analyzed and ranked NCDI conditions in several dimensions, including the burden of disease, severity, and disability, using data from the GBD study.^23^ The overall burden of disease of each condition at the national level was measured by disability-adjusted life-years (DALYs). Severity was calculated using the average years of life lost (YLLs) per death, and disability was calculated by years lived with disability (YLDs) per prevalent case. The age-standardized DALY rate per 100,000 population was compared for each condition to high-income country rates as a reference standard. A total of 190 NCDI conditions from the GBD database were analyzed using these 4 metrics, and a summary score was provided to each condition according to an average of the ranking quartiles. Each of the 4 metrics was weighted according to its relative importance as determined by each commission. The 50 conditions with the highest priority summary scores were then reviewed by a subcommittee of the national commission. Commissioners then selected conditions that they believed (1) contribute significantly to adverse health and economic consequences in their respective country, (2) could be feasibly and effectively controlled in their local context, and (3) were aligned with or complementary to existing national policies and strategic plans.

Information regarding evidence-based and cost-effective health-sector interventions was obtained from the Disease Control Priorities Project, which produced model lists of essential health-sector interventions and essential intersectoral health policies for the Disease Control Priorities, 3^rd^ Edition (DCP3) publication. DCP3 recommended 21 packages consisting of a total of 218 health-sector interventions to achieve essential universal health coverage (EUHC) in LLMICs based on key intervention metrics, including cost-effectiveness, financial risk protection, and equity scores (Supplement Table 2).^24^ The 65 interventions pertaining to NCDI conditions recommended as part of EUHC were reviewed by the national commissioners and evaluated for (1) alignment with stated NCDI priority conditions; (2) feasibility and desirability in the national context, and (3) cost-effectiveness, financial risk protection, and equity scores as assessed by DCP3.^25^ Commissions conducted customized deliberative processes to select a final set of interventions. Several commissions also considered additional interventions suggested by commissioners using locally available data (not included in costing estimates). In Ethiopia, the commission considered an expanded set of 235 interventions consisting of DCP3-recommended interventions as well as locally customized interventions.^26^

The commissions estimated the costs of their recommended interventions using methods and data that built on the cost modeling done for DCP3.^27^ In brief, costs were estimated as a function of: (1) the number of beneficiaries requiring each intervention (derived from GBD incidence, prevalence, or population estimates); (2) unit costs for each intervention (derived from published literature and extrapolated to different countries based on variation in labor and capital costs); and (3) proportion of the population “covered” by the intervention. Each intervention was assigned a current coverage, estimated from existing data sources or expert opinion (from the commissioners) if local data were not available. The commissions then assigned a feasible target coverage for each intervention within a timeframe of 5 to 10 years. Finally, the total annual cost of implementing each intervention at the additional coverage increment was calculated as the product of (1), (2), and (3). The commission in Ethiopia used an alternative method to estimate the cost of prioritized recommendations based on bottom-up costing through the use of the One Health tool, described in greater detail elsewhere.^26^

In each country, findings and recommendations were synthesized and disseminated to national stakeholders. The target audience for commission recommendations included government officials (national and subnational level), clinical leaders, implementation partners, donors, patient advocacy groups, local media, and the general public. Dissemination occurred through the publication of findings and recommendations, public launch events, media coverage, social media, advocacy meetings, and the NCDI Poverty website (Supplement Table 1). To further enhance awareness and local understanding of these conditions, commissions were additionally invited to develop video documentary narratives of young individuals living with severe NCDIs in populations of extreme poverty. Patients were selected for the documentary narratives as determined by each commission.

### Design and Methodology of Evaluation

From April to November 2019, the *Lancet* NCDI Poverty Commission Secretariat conducted a 2-part evaluation to understand how commissions influenced national-level dialogue and policies. This evaluation consisted of a semistructured online survey and phone-based qualitative interview. Each of the commissions in Round I was invited to nominate a key informant (generally a commission chair or coordinator) to complete the online survey. Respondents were asked a series of open-ended questions related to their respective commission’s work in 11 thematic areas corresponding to major objectives of the *Lancet* NCDI Poverty Commission framework.^5^ The survey was administered through the Qualtrics platform without time limitation. Respondents were encouraged to consult with other members of their national commissions in developing their responses to the online survey.

After completing the online survey, respondents were invited to participate in a follow-up interview in which the respondent could provide in-depth, detailed elaborations on survey responses.

Online and interview responses were entered into Dedoose version 8.3.35. Each transcript was coded independently by the 2 researchers. The number of respondents reporting positive outcomes on the online survey or interview was tabulated by thematic area.

The online survey and qualitative interviews were approved by the Harvard University Longwood Medical Area Institutional Review Board. All participants were provided a standardized consent form and asked to provide consent before beginning the data collection. Consent was provided electronically for the online survey and verbally for the interviews.

## RESULTS

### Commission Countries and Characteristics

In Round I, commissions were established in 9 countries including Afghanistan, Ethiopia, Haiti, Kenya, Liberia, Malawi, Mozambique, Nepal, and Tanzania (Table 1). In Rwanda, a research group was formed to undertake this analysis rather than a formal commission. In Round II, commissions were established in an additional 5 countries including Madagascar, Sierra Leone, Uganda, Zambia, and Zimbabwe, and 1 subnational region (Chhattisgarh State, India). Two countries (Rwanda and Zambia) opted to not use the nomenclature of “commission” formally and instead used the terms “group” and “task force”, respectively. Although formally established, the commission in Madagascar had not yet begun activities as of July 2020. Twenty-two individuals were nominated as commissioners or official group participants for each commission. As of July 2020, 7 commissions had completed the prioritization exercise and cost estimation analysis (Phase 1B) and an additional 5 commissions were in the process of completing this analysis. Additionally, 80 patient narratives were developed in video documentary format across 5 countries representing a range of NCDI conditions, including type I diabetes, rheumatic heart disease, chronic kidney disease, breast cancer, cervical cancer, neuroblastoma, childhood leukemia, schizophrenia, and disability due to traumatic injury.^28^

**TABLE 1.** Key Characteristics of States and Countries With Established National Noncommunicable Diseases and Injuries Poverty Commissions

	**Demographics**			**Finances** ^20^			**Human Resources** ^21^		**Commission Characteristics**		
**Country**	**Population (millions)** ^1^	**Percent Living in Poorest Billion** ^16^ **^,c^**	**Population Living in Poorest Billion (millions)** ^1^ **^,^** ^5^ **^,^** ^16^	**Gross Domestic Product per Capita (US$), 2017**	**Current Health Expenditure per Capita (US$), 2017**	**General Government Health Expenditure per Capita (US$), 2017**	**Physicians per 10,000 Population (year)**	**Nurses/Midwives per 10,000 Population (year)**	**Month and Year of Commission Establishment** ^a^	**Number of Commissioners**	**Priority Setting Conducted as of July 2020 **
Afghanistan	32.9	25.1	8.3	569.9	67.1	3.4	2.8 (2016)	1.8 (2017)	April 2018	22	No
Chhattisgarh State,^b^ India	27.7	15.8	4.4						March 2019	20	No
Ethiopia	102.9	81.5	83.8	721.2	25.3	6.3	0.8 (2018)	7.1 (2018)	Aug 2016	18	Yes
Haiti	11.8	39.0	4.6	776.0	62.4	7.4	2.3 (2018)	6.8 (2018)	Dec 2016	28	Yes
Kenya	48.3	38.9	18.8	1595.2	76.6	32.7	1.6 (2018)	11.7 (2018)	Nov 2016	25	Yes
Liberia	4.7	48.3	2.3	694.0	56.6	9.7	0.4 (2015)	5.3 (2018)	Jan 2017	23	Yes
Madagascar	26.1	65.2	17.0	448.4	24.7	11.6	1.8 (2014)	1.5 (2018)	July 2019		No
Malawi	17.2	48.5	8.3	334.4	32.3	9.9	0.4 (2018)	4.4 (2018)	Nov 2016	23	Yes
Mozambique	30.0	65.1	19.6	426.4	21.1	6.3	0.8 (2018)	6.8 (2018)	June 2017	14	Yes
Nepal	29.9	12.6	3.8	862.8	47.9	10.7	7.5 (2018)	31.1 (2018)	Nov 2016	20	Yes
Rwanda	12.6	49.2	6.2	748.7	49.2	16.9	1.3 (2018)	12.0 (2018)	March 2017	17	No
Sierra Leone	7.8	62.2	4.9	494.8	66.4	9.1	0.3 (2011)	2.2 (2016)	Aug 2018	38	Yes
Tanzania	54.0	56.2	30.4	930.4	33.9	14.7	0.1 (2016)	5.8 (2017)	Sept 2016	7	Yes
Uganda	39.1	58.4	22.8	621.0	38.9	6.0	1.7 (2017)	12.4 (2018)	Jan 2019	26	Yes
Zambia	17.4	46.7	8.1	1513.3	67.6	26.1	11.9 (2018)	13.4 (2018)	Aug 2018	22	Yes
Zimbabwe	14.7	28.6	4.2	1659.9	110.1	56.9	2.1 (2014)	19.3 (2018)	Aug 2018	25	Yes
Total or Averages^c^	477.0	51.9	247.4	826.4	52.0	15.2	2.4	9.4		328	

^a^ Date of endorsement by formal communication from Ministry of Health or first official commission meeting, whichever came first.

^b^ State-level indicators not available from sources listed.

^c^ Average of “percent living in poorest billion” is weighted average. All other averages are unweighted averages.

### Commissions’ Findings of NCDI Burden of Disease and Expenditure (Phase 1a)

On average across the 16 countries and states, 45.2% of DALYs were attributed to NCDIs (range 33.5–68.6%; Table 2), and 55.1% of DALYs due to NCDIs occurred before age 40 years (range: 36.4–62.9%). Additionally, 60.2% of the NCD DALYs across this group of countries were associated with NCD conditions other than those comprising the 4 disease categories included in the global NCD action plan (cardiovascular diseases, chronic respiratory diseases, diabetes, and cancer). When considering an expanded “5 by 5” framework that includes mental health and substance use disorders, 49.2% of NCD DALYs were from other conditions. In terms of injuries, 73.7% of DALYs due to injuries across this set of countries were due to injuries other than road traffic injuries (data not shown here).

**TABLE 2.** Baseline National NCDI Poverty Commission Findings on the Proportion, Severity, Diversity, and Expenditure on NCDIs^a^

	**Estimated Burden of NCDIs** ^1^			**General Government Health Expenditure on NCDIs (2017)** ^20^		**External Health Expenditure on NCDIs (2017)** ^20^	
	**% DALYs from NCDIs**	**% NCDI DALYs Occurring Before Age 40 years**	**% NCD DALYs not due to 5x5 Conditions** ^b^	**% General Government Health Expenditure on NCDs**	**% General Government Health Expenditure on injuries**	**% External Health Expenditure on NCDs**	**% External Health Expenditure on injuries**
Afghanistan	55.2	61.8	46.3	38.4	2.5	8.8	4.8
Chhattisgarh State, India^c^	62.3	37.4	41.9				
Ethiopia	40.2	59.7	53.4	19.5	5.3	0.7	0.1
Haiti	63.3	51.8	41.5	20.6	7.7	4.8	5.7
Kenya	43.4	53.1	52.3	4.3	3.2	0.9	0.6
Liberia	37.6	56.4	54.1	17.8	1.3	8.6	0.6
Madagascar	38.9	55.1	45.0				
Malawi	36.2	58.0	51.4	11.4	8.9	7.2	5.6
Mozambique	33.5	59.1	50.2				
Nepal	68.6	36.4	41.5	50.5	1.1	13.5	1.4
Rwanda	49.5	57.1	53.2				
Sierra Leone	33.9	60.2	54.2				
Tanzania	43.4	61.9	56.8	12.9	2.1	2.2	0.5
Uganda	36.9	62.9	51.7	26.0	4.0	1.8	0.3
Zambia	37.5	60.6	53.6	12.9	8.6	12.9	
Zimbabwe	42.5	50.5	40.1				
**Average (unweighted)**	**45.2**	**55.1**	**49.2**				

Abbreviations: DALY, disability adjusted life year; NCDI, noncommunicable disease and injury.

^a^ Gray boxes indicate national health account data is unavailable or not disaggregated for NCDIs.

^b^ ‘5x5 conditions refers to Global Burden of Disease categories of cardiovascular diseases, diabetes, cancer, chronic respiratory diseases, and mental health disorders.

^c^ State-level indicators not available from sources listed.

Across 16 countries and states, 60.2% of the NCD DALYs were associated with NCD conditions other than those comprising the 4 disease categories in the global NCD action plan.

Ten of the 15 countries (not including Chhattisgarh state) had available estimates for domestic government and external expenditures for NCDs and injuries. In these countries, the proportion of government health expenditure for NCDIs as a proportion of general government health expenditures ranged considerably, from 4.3%–50.5% for NCDs and 1.1%–8.9% for injuries. The proportion of external expenditures was consistently lower for NCDIs, from 0.7%–12.9% for NCDs and 0.1%–5.7% for injuries. In Liberia, 74.7% of expenditures for NCDIs were out-of-pocket, exceeding the proportion of out-of-pocket expenditure for other disease areas, such as malaria (60%), TB (48%), HIV/AIDS (30%), and reproductive, maternal, neonatal, child, and adolescent health (19%).^29^ In Ethiopia, 68% of expenditures for NCDIs were out-of-pocket.^26^

### Commissions’ Recommendations on Prioritization for NCDI Conditions and Health-Sector Interventions (Phase 1b)

As of July 2020, 7 commissions completed a prioritization exercise for NCDI disease conditions and health-sector interventions (Unpublished Haiti NCDO Poverty Commission report).^26^^,^^29^^–^^33^ The commission in Ethiopia conducted a prioritization exercise for an expanded set of NCDI health interventions but not for disease conditions. Prioritization exercises had also been initiated in Mozambique, Sierra Leone, Uganda, Zimbabwe, and Zambia, though they were not yet completed and subsequently not included here. After compiling and analyzing data on the burden, severity, disability, and equity metrics from GBD, and undergoing a commission review and validation, the 6 commissions that had completed the process and had prioritized among NCDI conditions selected between 14 and 48 conditions for prioritization (Table 3). Overall, 75 of 211 total NCDI conditions were selected by at least 1 commission. Fifteen conditions were selected by all 6 commissions. These conditions included asthma, breast cancer, cervical cancer, diabetes mellitus type 1, diabetes mellitus type 2, epilepsy, hypertensive heart disease, intracerebral hemorrhage, ischemic heart disease, ischemic stroke, major depressive disorder, motor vehicle road injuries, rheumatic heart disease, sickle cell disorders, and subarachnoid hemorrhage. An additional 12 conditions were selected by at least 4 of the 6 commissions.

**TABLE 3.** Results of National NCDI Poverty Commissions Prioritization of NCDI Conditions and NCDI Health-Sector Interventions

	**Number Of Prioritized Conditions**	**Number Of Recommended Interventions**	**Incremental Intervention Coverage Increase, %**	**Annual Incremental Expenditure to Provide Target Coverage for Recommended Interventions (mil US$)**	**Annual Incremental Cost per Capita to Provide Target Coverage Of Recommended Interventions (US$)**	**Percentage of Current Total Health Expenditure per Capita, %**	**Percentage of Current Gross Domestic Product per Capita, %**
Ethiopia^a^	N/A	90	30	550	4.70	16.8	0.6
Haiti	36	36	25	69.1	6.44	9.7	0.8
Kenya	14	34	20-70	520.1	11.97	17.1	0.7
Liberia	19	33	30	29.5	9.21	13.8	2.1
Malawi	38	54	0-50	236.7	13.70	34.2	4.0
Nepal	25	23	30	250.2	8.76	22.0	1.4
Tanzania	48	53	30	702.9	12.26	35.6	1.3

Abbreviation: N/A, not applicable; NCDI, noncommunicable disease and injury.

^a^ In Ethiopia, the commission considered an expanded set of 235 interventions consisting of DCP3-recommended interventions as well as locally customized interventions.

Six commissions considered 65 health-sector NCDI-focused interventions recently recommended as a package for EUHC. Thirty-two interventions were selected by a majority (>/=4) of the commissions, including 18 that were selected by all commissions (Table 4). Among these 32 interventions, 19 targeted the health center level, 6 targeted first-level hospitals, 4 at referral/specialty hospitals, 2 for population-level mass media messages, and 1 at the community level. These interventions included 4 surgical “packages” at various levels of the health system and packages for palliative care and rehabilitation services. Of the 18 interventions selected by all commissions, 17 were recommended as “high-priority” EUHC interventions by DCP3. The commission in Ethiopia conducted the prioritization exercise considering all 235 health-sector interventions they had identified and selected 90 interventions as high-priority. Including the commission in Ethiopia, the annual cost across the 7 commissions of the prioritized interventions at the target intervention coverage assigned by each commission ranged from US$4.70–US$13.70 per capita. This amount of per capita expenditure ranged from 9.7% (Haiti) to 35.6% (Tanzania) of the most recently reported total health expenditure per capita and from 0.6% (Ethiopia) to 4.0% (Malawi) of gross domestic product per capita at the time of the respective analyses.

**TABLE 4.** Most Commonly Selected Conditions and Corresponding Prioritized Health Sector Interventions by 6 Commissions Conducting Prioritization Exercises by Both Disease Conditions and Health Sector Interventions (Displaying Conditions and Interventions Selected by >/= 4 Commissions)

**Disease Condition Category**	**Selected Condition (No. of Commissions)**	**Corresponding Health Sector Intervention (No. of Commissions)**
Cardiovascular	Hypertensive heart disease (6) ischemic heart disease (6) ischemic stroke (6) intracerebral hemorrhage (6) subarachnoid hemorrhage (6)	Long term management of ischemic heart disease, stroke, and peripheral vascular disease with aspirin, beta blockers, angiotensin-converting enzyme inhibitors, and statins (as indicated), for secondary prevention (6) Mass media messages concerning healthy eating or physical activity (6) Use of aspirin in case of suspected myocardial infarction (6) Medical management of acute heart failure (6) Medical management of chronic heart failure with diuretics, beta-blockers, ace-inhibitors, and mineralocorticoid antagonists (6) Opportunistic screening for hypertension for all adults, with treatment decisions guided by absolute cardiovascular disease risk (4) Screening and management of hypertensive disorders in pregnancy (4)
	Rheumatic heart disease (6)	Secondary prophylaxis with penicillin for rheumatic fever or established rheumatic heart disease (6) Treatment of acute pharyngitis in children to prevent rheumatic fever (6) Heart failure interventions as above cardiovascular disease
	Congenital heart anomalies (4)	No specific interventions
Diabetes	Diabetes (type 1 and 2) (6)	Prevention of long-term complications of diabetes through blood pressure, lipid, and glucose management as well as consistent foot care (6) Screening for diabetes in pregnant women (6)
Respiratory	Asthma (6) Chronic obstructive pulmonary disease (5)	Mass media messages concerning use of tobacco and alcohol (6) Low-dose inhaled corticosteroids and bronchodilators for asthma and for selected patients with chronic obstructive pulmonary disease (5) Management of acute exacerbations of asthma and chronic obstructive pulmonary disease using systemic steroids, inhaled beta-agonists, and, if indicated, oral antibiotics and oxygen therapy (5) Tobacco cessation counseling and use of nicotine replacement therapy in certain circumstances (4)
	Breast cancer (6)	Treat early stage breast cancer with appropriate multimodal approaches, including generic chemotherapy, with curative intent, for cases that are referred from health centers and first-level hospitals following detection using clinical examination (6) Palliative care and pain control services^a^ (5)
	Cervical cancer (6)	Treatment of early-stage cervical cancer (6) Opportunistic screening for cervical cancer using visual inspection or human papillomavirus DNA testing and treatment of precancerous lesions with cryotherapy (5) School-based human papillomavirus vaccination for girls (5)
	Non-Hodgkin lymphoma (5)	Treat selected early-stage childhood cancers with curative intent in pediatric cancer units/hospitals (6)
Sickle cell disease	Sickle cell disease (6)	In settings where sickle cell disease is a public health concern, universal newborn screening followed by standard prophylaxis against bacterial infections and malaria^a^ (6)
Chronic kidney disease	Due to glomerulonephritis (4) Due to hypertension (4)	Treatment of hypertension in kidney disease, with use of angiotensin-converting enzyme inhibitors or angiotensin receptor blockers in albuminuric kidney disease (5)
Liver cirrhosis	Cirrhosis due to hepatitis B virus (5) Alcoholic cirrhosis (5)	Mass media messages concerning use of tobacco and alcohol (6)
Acute abdominal conditions	Paralytic ileus and intestinal obstruction (4)	Basic first-level hospital surgical services^a^ (6) Expanded first-level hospital surgical services^a^ (4)
Epilepsy	Epilepsy (6)	Management of epilepsy using generic anti-epileptics (6)
Mental health	Depression (5) Anxiety (4)	Management of depression and anxiety disorders with psychological and generic antidepressant therapy (6) Management of schizophrenia using generic anti-psychotic medications and psychosocial treatment (5) Management of bipolar disorder using generic mood-stabilizing medications and psychosocial treatment (4)
Injuries	Motor vehicle road injuries (6) Pedestrian road injuries (4) Burns (4)	Basic outpatient surgical services^a^ (4) Basic first-level hospital surgical services^a^ (6) Expanded first-level hospital surgical services^a^ (4) Specialized surgical services^a^ (6) Basic rehabilitation services^a^ (5)

^a^ Indicates a set of interventions.

The annual cost across the 7 commissions of the prioritized interventions at the target intervention coverage assigned by each commission ranged from US$4.70–US$13.70 per capita.

## RESULTS OF EVALUATION

**FIGURE 2:**
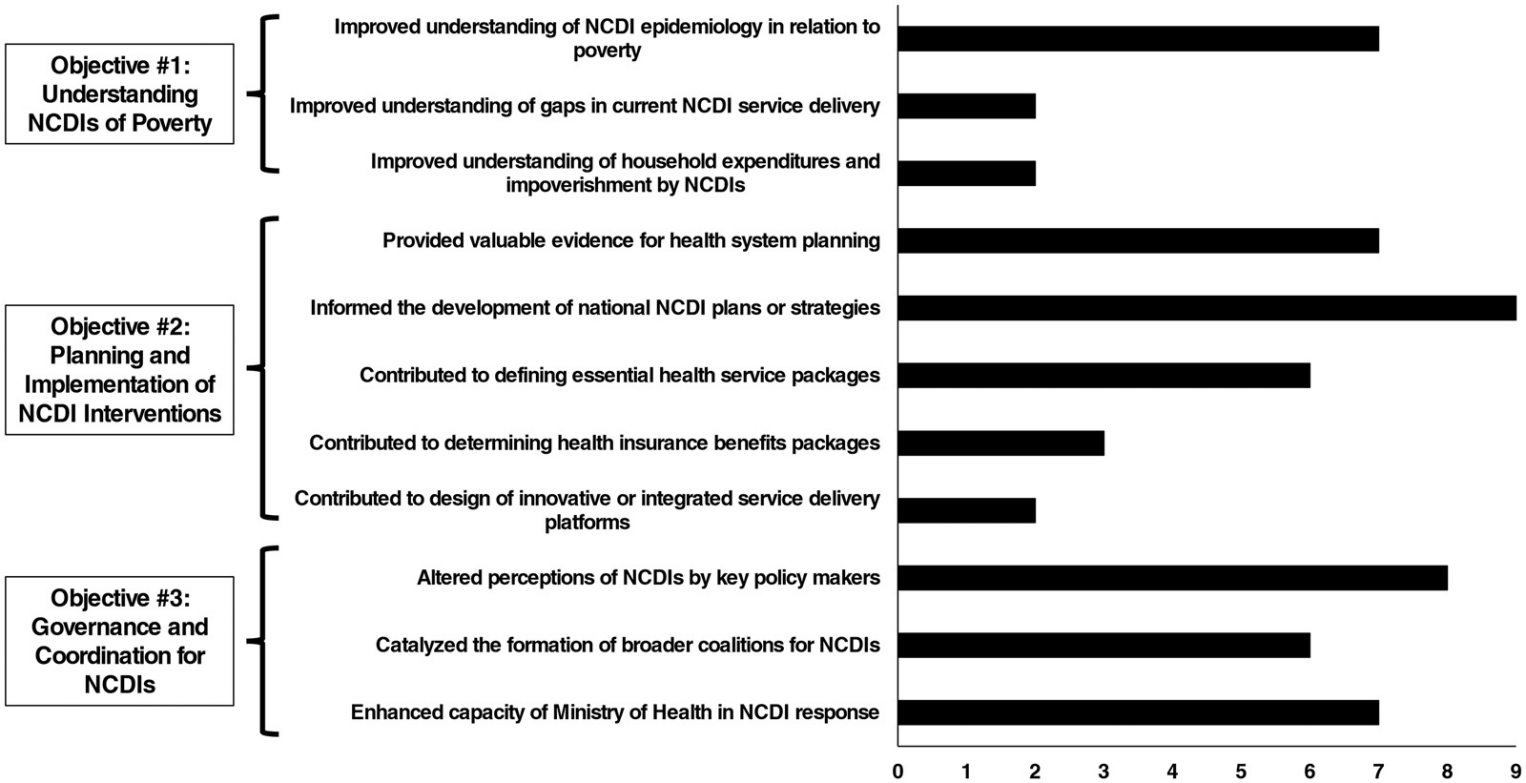
Frequency of National Noncommunicable Diseases and Injuries Poverty Commissions Reporting Key Outcomes in Evaluation Surveys and Qualitative Interviews, by Thematic Area (n=9) Abbreviation: NCDI, noncommunicable diseases and injuries.

Responses were received from each of the 9 commissions formally established in Round 1. Findings were categorized according to the 3 overall NCDI Poverty Commission objectives: “Understanding NCDIs of Poverty,” “Planning and Implementation of NCDI Interventions,” and “Governance and Coordination for NCDIs” (Figure 2). Respondents provided key contextual information on successes achieved and challenges experienced by the national commissions (key aspects highlighted in Supplement Tables 3 and 4). Most respondents reported that their respective commissions improved their understanding of NCDI epidemiology in relation to poverty (n=7). However, only a minority of respondents reported improvements in understanding of gaps in current NCDI service delivery (n=2) or household expenditures and impoverishment by NCDIs (n=2). All country respondents indicated that their respective commissions had informed the development of national NCDI plans or strategies, and most reported provision of valuable evidence for health system planning (n=7) and contribution to defining essential health service packages (n=6). Several respondents reported contributions by their respective commissions for health insurance benefits packages (n=3) and the design of innovative or integrated services platforms (n=2). There was a strong theme of commissions supporting the governance of coordination of NCDIs, with most respondents indicating that their commissions altered perceptions of NCDIs by key policy makers (n=8), catalyzed the formation of broader coalitions for NCDIs (n=6), and enhanced capacity of the Ministry of Health in their NCDI response (n=7).

## DISCUSSION AND NEXT STEPS

We have described the establishment, structure, and findings of national- and state-level NCDI Poverty Commissions established in 16 LLMICs with significant populations living in extreme poverty. The NCDI Poverty Commissions represent a coordinated effort to quantify the comprehensive burden of NCDIs and provide evidence-based recommendations on health-sector interventions to strengthen NCDI services under commitments to UHC.

The NCDI Poverty Commissions represent a coordinated effort to quantify the comprehensive burden of NCDIs and provide evidence-based recommendations on health-sector interventions to strengthen NCDI services under commitments to UHC.

Current expenditure on NCDIs reported by the commissions varied widely by country, though these findings may be subject to high variability and low reliability of national health accounts.^34^ The commissions estimated that to implement the prioritized health-sector NCDI interventions for a substantial target population would require an additional US$4.70–US$13.70 per capita, or approximately 9.7%–35.6% of current total health expenditure (or 0.6%–4.0% of current gross domestic product). The commissions in Malawi and Tanzania established more expansive criteria for selecting health-sector interventions to drive dialogue and advocacy for health system expansion and investment. Commissions in Nepal and Kenya opted for a more targeted approach to selecting interventions to focus advocacy on key interventions currently lacking in their respective health systems. The overall EUHC health benefits package was previously estimated to cost US$79 per person at 80% population coverage in low-income countries, representing an additional investment of 8.0% of the 2015 gross national income.^27^ Interventions to reduce mortality from NCDIs comprised at least 37.6% of these EUHC estimated costs, not including substantial costs of interventions targeting the reduction of disability from NCDIs. The interventions prioritized and costed by the commissions represent a more conservative incremental intervention coverage target (i.e., 30%) and focus on a smaller subset of NCDI interventions, though still include a wide range of medical, surgical, mental health, palliative care, and rehabilitation services, as well as indirect costs for laboratory services, infrastructure, administration, and management.

A recent analysis by the *Lancet* NCDI Poverty Commission showed that this level of additional expenditure for NCDIs may be possible within domestic financing under conditions of strong economic growth, increased tax revenues, and greater proportional allocation of government expenditures to health consistent with recent targets.^5^^,^^35^ However, such conditions may be unlikely in most LLMICs, particularly in the context of severe economic challenges incurred during the coronavirus disease (COVID-19) pandemic, and external development assistance will be required to finance this critical set of interventions.

Most commissions identified opportunities to integrate prioritized health-sector interventions into existing platforms to improve efficiencies and facilitate operationalization. Decentralization may rely on training and task optimization for nurses and other mid- to low-level cadres of the health workforce, and clustering of services based on intervention properties.^36^^,^^37^ Further efforts to more accurately define additional interventions within national contexts are needed to inform the development of essential health service packages, health insurance coverage schemes, and national financing priorities. Intersectoral policies were not explicitly prioritized and costed by the commissions and require further consideration given their vital role in the prevention of NCDIs.

The urgent need for strong integrated health systems to mitigate and address the adverse effects of NCDs in LLMICs has been magnified by the COVID-19 pandemic.

The urgent need for strong integrated health systems to mitigate and address the adverse effects of NCDs in LLMICs has been magnified by the COVID-19 pandemic.^38^^,^^39^ NCDI Poverty Commissions provide an opportunity for local definition and accountability for a context-specific and locally relevant NCDI agenda under rapidly evolving epidemiology and health system constraints. National commissioners highlighted the impact of these commissions in changing the traditional perceptions and framing of NCDIs with a focus on severe conditions affecting poor, rural populations at young ages. The priority-setting process utilized in this analytic framework was additive and contributory to national strategic and operational planning efforts and packages of essential services, core components to health system readiness, and achievement of commitments to UHC. The commissions provided a productive forum for policy makers to meet with academic and implementing stakeholders to critically analyze and consider available data and formulate evidence-based recommendations. National commissioners highlighted the role of commissions in strengthening in-country public-sector capacity in the response to NCDIs and expanding perceptions of NCDIs by key policymakers. Evaluation of a group of these commissions demonstrated substantial contributions of the commissions in informing national efforts in strategic and health system planning for NCDIs as well as the understanding and capacity of key governance structures for the NCDI response at a national level.

The collective experience of the commission processes and findings presented here has informed the establishment of the NCDI Poverty Network.^40^ The NCDI Poverty Network aims to catalyze technical and financing partnerships to improve the understanding, awareness, investment, and outcomes for individuals living with NCDIs in settings of extreme poverty. National commissions will further assess the current availability and organization of these interventions in both public and private-sector facilities, with a particular focus on integrating and optimizing human resource availability and capacities. In addition, national commissions will play a key role in defining, piloting, and scaling potential facility-based delivery models for severe conditions impacting younger populations, such as “PEN-Plus.”^41^ The NCDI Poverty Network will aim to promote cross-border collaboration for an expanded NCDI agenda, build programmatic and research capacity, and establish global financing partnerships to support the availability and coverage of NCDI interventions. These will be necessary steps to fulfill national commitments to UHC for populations facing the double burden of extreme poverty and disproportionately suffering from the physical and economic consequences of a highly diverse array of NCDIs.

## Supplementary Material

21-00035-Gupta-Supplement.pdf

## Notes

**Peer Reviewed**

**First published online:** August 3, 2021.
