# Supplementary material for: Prioritizing Health-Sector Interventions for Noncommunicable Diseases and Injuries in Low- and Lower-Middle Income Countries: National NCDI Poverty Commissions
Source: Glob Health Sci Pract. 2021 Sep 30;9(3):626–39. doi: 10.9745/GHSP-D-21-00035 (PMC8514044; doi:10.9745/GHSP-D-21-00035)
Supplement: 21-00035-Gupta-Supplement.pdf [file 21-00035-Gupta-Supplement.pdf]

Supplement to: Gupta N, Mocumbi A, Arwal SH, et al. Priority-setting of health-sector interventions for noncommunicable diseases and injuries to achieve universal health coverage low- and lower-middle income countries. *Glob Health Sci Pract*. 2021;9(3). https://doi.org/10.9745/GHSP-D-21-00035

Supplement Table 1. Additional Information Regarding National NCDI Poverty Commission Composition and Data Sources

Country	Co-chair Affiliations and Host Organizations	Number of articles retrieved/number of articles meeting inclusion criteria for relevance	Data Sources Utilized	Date of National Dissemination of Findings and Recommendations
Round I				
Afghanistan	Afghanistan Ministry of Public Health	3513/34	Literature Review, Policy Review, GBD 2017	April 2019
Ethiopia	Ethiopia Ministry of Heath	948/206*	Literature Review, Policy Review, GBD 2016, STEPs 2015, SPA Survey Plus 2014, SARA 2016, DHS 2016, Household Health Service Utilization and Expenditure Survey 2015/16, NHA 2013/14, qualitative patient interviews (“Voices of NCDI Poverty”)	Nov 2018
Haiti	Ministère de la Santé Publique et de la Population, Fondation Haïtienne de Diabète et de Maladies Cardio-Vasculaires, Zanmi Lasante	N/A	Literature Review, Policy Review, DHS 2016-17, Haitian Système d'Information Sanitaire National Unique, Hôpital Universitaire de Mirebalais facility records, GBD 2016, SPA 2013, qualitative patient interviews (“Voices of NCDI Poverty”), OPHI	
Kenya	Kenya Ministry of Health, African Institute for Health and Development	2429/104	Literature Review, Policy Review, GBD 2016, Siaya HDSS, Nairobi HDSS, STEPs 2015, SARA 2013, DHS 2014, NHA 2015/16, OPHI	July 2018
Liberia	Ministry of Health Liberia, Partners In Health Liberia	542/51	Literature Review, Policy Review, GBD 2016, STEPs 2011, SARA 2016, DHS 2013, NHA 2013/14, Household Income and Expenditure Survey 2016, qualitative patient interviews	Oct 2018
Malawi	Malawi Ministry of Health, Partners In Health Malawi	910/130	Literature Review, Policy Review, GBD 2015, STEPs 2011, SPA 2013-14, DHS 2015-16, NHA 2014/15, Integrated Household Survey 4 2016-17, MOH Resource Mapping FY15/16, Malawi Epidemiology and Intervention Research Unit, Central Medical Stores Trust, qualitative patient interviews (“Voices of NCDI Poverty”)	Aug 2018
Mozambique	Mozambique Institute for Health Education and Research	308/66	Literature Review, Policy Review, GBD 2015, STEPs 2015, Reference Hospitals of Maputo and Matola, key informant interviews, OPHI, DHS 2016	June 2018
Nepal	Ministry of Health and Population of Nepal, Manmohan Cardiothoracic Vascular and Transplant Center, Dhulikhel Hospital – Kathmandu University Hospital	3035/229	Literature Review, Policy Review, GBD 2015, STEPs 2013, SPA 2015, DHS 2016, Nepal Health & Living Standards Survey 2011, qualitative patient interviews (“Voices of NCDI Poverty”)	March 2018
Rwanda*	Rwanda Ministry of Health & Rwanda Biomedical Center	664/74	Literature Review, Policy Review, GBD 2016, STEPs 2013, DHS 2015, Rwanda Integrated Household Living Conditions Survey 4, Rwanda Biomedical Center NCD Division facility assessment and mentorship data, National budget 2017-18	
Tanzania	Tanzanian Ministry of Health, Community Development, Gender, Elderly and Children, National Institute for Medical Research	1161/345	Literature Review, Policy Review, GBD 2017, STEPs 2012, SPA 2014, DHS 2015-16, NHA 2015/16, qualitative patient interviews	Nov 2019
Round II				
Chhattisgarh State, India	National Health Mission, Chhattisgarh		Literature Review, Policy Review, National Sample Survey, State Health Resource Center surveys, National Family Health Survey, Rashtriya Bal Swasthya Karyakram	
Madagascar	Ministere de la Sante Publique Madagascar, PIVOT		Not initiated	
Sierra Leone	Sierra Leone Ministry of Health and Sanitation, Partners In Health Sierra Leone		Literature Review, Policy Review, HMIS, SARA 2017, Sierra Leone Basic Package of Essential Health Services 2015-2020	
Uganda	Uganda Ministry of Health, Uganda Initiative for Integrated Management of Noncommunicable Disease		Literature Review, Policy Review, HDSS Iganga-Mayuge, HMIS, SARA 2018, NHA 2018	
Zambia	Zambia Ministry of Health, Centre for Infectious Disease Research in Zambia		Literature Review, Policy Review, STEPs 2017, Living Conditions Monitoring Survey 2015, DHS 2018, HMIS, NHA	
Zimbabwe	Zimbabwe Ministry of Health and Child Care, SolidarMed Zimbabwe		Literature Review, Policy Review, SARA 2015, DHS 2015, NHA	

*In Rwanda, a research group was established rather than a commission. DHS=Demographic Health Survey; GBD=Global Burden of Disease; HDSS=Health Demographic Surveillance Site; HMIS=Health Management Information System; NHA=National Health Account; OPHI=Oxford Poverty & Human Development Initiative; SARA=Service Availability and Readiness Assessment; SPA=Service Provision Assessment; STEPs=WHO Stepwise Approach to Surveillance.

Supplement to: Gupta N, Mocumbi A, Arwal SH, et al. Priority-setting of health-sector interventions for noncommunicable diseases and injuries to achieve universal health coverage low- and lower-middle income countries. *Glob Health Sci Pract*. 2021;9(3). https://doi.org/10.9745/GHSP-D-21-00035

Supplement Table 2. Essential UHC (EUHC) Health Sector Interventions for NCD and Injury Related Conditions and Assigned Intervention Properties*

Intervention	Condition	Included in DCP3 High-Priority Package	Platform	Cost-Effectiveness Score	Financial Risk Protection Score	Equity Score	Average Per Beneficiary Cost for LICs (USD)
Screening and brief intervention for alcohol use disorders	Alcoholic cirrhosis	No	Health center	3	2	1	3.61
Education campaigns for the prevention of gender-based violence	Assault/Violence	Yes					0.22
Post gender-based violence care including, counseling, provision of emergency contraception, and rape-response referral (medical and judicial)	Assault/Violence	Yes					3.58
Low-dose inhaled corticosteroids and bronchodilators for asthma and for selected patients with COPD	Respiratory	No	Health Center	1	3	1	50.61
Management of acute exacerbations of asthma and COPD using systemic steroids, inhaled beta-agonists, and, if indicated, oral antibiotics and oxygen therapy	Respiratory	No	First-level Hospital	1	4	1	127.18
Self-management for obstructive lung disease to promote early recognition and treatment of exacerbations	Respiratory	No					5.19
Tobacco cessation counseling, and use of nicotine replacement therapy in certain circumstances	Respiratory; Cardiovascular	No	Health Center	4	2	1	13.28
Mass media messages concerning use of tobacco and alcohol	Respiratory; Cardiovascular; Alcoholic cirrhosis	No	Population	4	1	1	0.01
Treat early stage breast cancer with appropriate multimodal approaches, including generic chemotherapy, with curative intent, for cases that are referred from health centers and first-level hospitals following detection using clinical examination	Cancer - Breast	Yes	Referral and Specialty Hospitals	4	4	1	238.64
Opportunistic screening for cervical cancer using visual inspection or HPV DNA testing and treatment of precancerous lesions with cryotherapy	Cancer - Cervical	No	Health Center	3	3	1	4.00
School-based HPV vaccination for girls	Cancer - Cervical	Yes	Community	3	3	1	9.14
Treatment of early-stage cervical cancer	Cancer - Cervical	Yes	First-level Hospital	0	4	1	35.58
Treat early stage colorectal cancer with appropriate multimodal approaches, including generic chemotherapy, with curative intent, for cases that are referred from health centers and first-level hospitals	Cancer – Colorectal	Yes	Referral and Specialty Hospitals	3	5	1	238.64
Treat selected early-stage childhood cancers with curative intent in pediatric cancer units/hospitals	Cancer – Leukemias	Yes	Referral and Specialty Hospitals	2	5	2	492.78
Palliative care and pain control services	Cancer; Palliative Care	Yes	Health Center		4	1	1.37
Treatment of hypertension in kidney disease, with use of ACEi or ARBs in albuminuric kidney disease	Chronic Kidney Disease	No	Health Center	2	2	1	46.95
Provide iron and folic acid supplementation to pregnant women, as well as food/caloric	Congenital	No	Health Center	3	3	3	13.92

Supplement to: Gupta N, Mocumbi A, Arwal SH, et al. Priority-setting of health-sector interventions for noncommunicable diseases and injuries to achieve universal health coverage low- and lower-middle income countries. *Glob Health Sci Pract*. 2021;9(3). https://doi.org/10.9745/GHSP-D-21-00035

Intervention	Condition	Included in DCP3 High-Priority Package	Platform	Cost-Effectiveness Score	Financial Risk Protection Score	Equity Score	Average Per Beneficiary Cost for LICs (USD)
supplementation to pregnant women in food insecure households							
Combination therapy for persons with multiple risk factors to prevent CVD (primary prevention)	Cardivascular	No	Health Center	2	2	1	38.33
Long term management of IHD, stroke, and PVD with aspirin, beta blockers, ACEi, and statins (as indicated), for secondary prevention	Cardivascular	Yes	Health Center	2	2	1	64.39
Management for acute critical limb ischemia with unfractionated heparin and revascularization if available, with amputation as a last resort	Cardivascular	No					665.09
Mass media messages concerning healthy eating or physical activity	Cardivascular	Yes	Population	4	1	1	0.01
Opportunistic screening for hypertension for all adults, with treatment decisions guided by absolute CVD risk	Cardivascular	No	Health Center	1	1	1	1.48
Screening and management of hypertensive disorders in pregnancy	Cardivascular	No	Health center	1	3	3	1.08
Use of aspirin in case of suspected myocardial infarction	Cardivascular	Yes	Health Center	4	2	1	0.03
Use of community health workers to screen for CVRD using non-lab-based tools for overall CVD risk, improving adherence, and referral to primary health centers for continued medical management	Cardivascular	No					0.14
Use of percutaneous coronary intervention for acute myocardial infarction where resources permit	Cardivascular	No	Referral and Specialty Hospitals	3	4	1	900.00
Use of unfractionated heparin, aspirin, and generic thrombolytics in acute coronary events	Cardivascular	No	First-level Hospitals	2	4	1	531.66
Medical management of acute heart failure	Cardivascular	Yes	First-level Hospital	4	5	3	763.39
Medical management of chronic heart failure with diuretics, beta-blockers, ace-inhibitors, and mineralocorticoid antagonists	Cardivascular	Yes	Health Center	4	4	3	287.85
Management of depression and anxiety disorders with psychological and generic antidepressant therapy	Depression	Yes	Health Center	3	4	1	13.38
Mass media messages concerning sexual and reproductive health; and mental health for adolescents	Depression	No	Population	4	2	1	1.16
Diabetes self-management education	Diabetes	No					5.19
Diabetic retinopathy screening via telemedicine, followed by treatment using laser photocoagulation	Diabetes	No	Referral and Specialty Hospitals	3	2	1	15.31
Prevention of long-term complications of diabetes through blood pressure, lipid, and glucose management as well as consistent foot care	Diabetes	Yes	Health Center	4	2	1	84.42
Screening for diabetes in all high-risk adults	Diabetes	No	Health center	4	2	1	1.44
Screening for diabetes in pregnant women	Diabetes	Yes	Health center	1	3	3	9.31
Management of epilepsy using generic anti-epileptics	Epilepsy	Yes	Health Center	4	4	3	22.86

Supplement to: Gupta N, Mocumbi A, Arwal SH, et al. Priority-setting of health-sector interventions for noncommunicable diseases and injuries to achieve universal health coverage low- and lower-middle income countries. *Glob Health Sci Pract*. 2021;9(3). https://doi.org/10.9745/GHSP-D-21-00035

Intervention	Condition	Included in DCP3 High-Priority Package	Platform	Cost-Effectiveness Score	Financial Risk Protection Score	Equity Score	Average Per Beneficiary Cost for LICs (USD)
Elective surgical repair of common orthopedic injuries (e.g., meniscal and ligamentous tears) in individuals with severe functional limitation	Injuries	No	Referral and Specialty Hospitals				91.48
Basic first-level hospital surgical services	Injuries; Surgery	Yes	First-level Hospital				3.75
Basic outpatient surgical services	Injuries; Surgery	No	Health center				0.38
Basic rehabilitation services	Injuries; Surgery	Yes	First-level Hospital				1.99
Expanded first-level hospital surgical services	Injuries; Surgery	No	Referral and Specialty Hospitals				0.05
Specialized surgical services	Injuries; Surgery	Yes	Referral and Specialty Hospitals				0.09
Management of bipolar disorder using generic mood-stabilizing medications and psychosocial treatment	Mental Health	Yes	Health Center	2	4	2	153.27
Management of schizophrenia using generic anti-psychotic medications and psychosocial treatment	Mental Health	Yes	Health Center	2	4	2	82.57
Secondary prophylaxis with penicillin for rheumatic fever or established RHD	Cardiovascular (RHD)	Yes	Health center	0	1	1	0.07
Treatment of acute pharyngitis in children to prevent rheumatic fever	Cardiovascular (RHD)	Yes	Health Center	4	2	1	0.14
In settings where sickle cell disease is a public health concern, universal newborn screening followed by standard prophylaxis against bacterial infections and malaria	Sickle Cell	Yes	First-level Hospital	4	2	3	3.34

* Adapted from: "Annex 3F: Findings from the Appraisal of Essential UHC Interventions". In: *Disease Control Priorities* (third edition): Volume 9, *Disease Control Priorities*, edited by D. T. Jamison, H. Gelband, S. Horton, P. Jha, R. Laxminarayan, C. N. Mock, R. Nugent. Washington, DC: World Bank.

Supplement to: Gupta N, Mocumbi A, Arwal SH, et al. Priority-setting of health-sector interventions for noncommunicable diseases and injuries to achieve universal health coverage low- and lower-middle income countries. *Glob Health Sci Pract*. 2021;9(3). https://doi.org/10.9745/GHSP-D-21-00035

Supplement Table 3. Examples of Specific Outcomes and Impacts Reported by National NCDI Poverty Commissions, by Country

Afghanistan	-Strengthening NCD governance through core support for the Ministry of Public Health NCD Division (financial and technical support)<br>-Presentations of findings at the Afghanistan Health Results Conference (2019), French Medical Institute for Mothers and Children International Conference (2019), and Prince Mahidol Award Ceremony (2019)<br>-Participation in global training and advocacy efforts
Ethiopia	-Strengthened NCD governance and coordination through core support to the MOH NCD Division through seconded technical advisor<br>-Broadened national NCDI policy through inclusion of prioritized interventions to the Ethiopia Health Service Package<br>-Informed the development of the Ethiopia National NCD Strategic Plan<br>-Catalyzed the establishment of a multi-sectoral NCD Committee<br>-Built national NCDI coalition, awareness, and understanding through broad stakeholder engagement<br>-Identified and explored integrated service delivery models<br>-Participation in global advocacy and network building, including the UN General Assembly (2018)
Haiti	-Strengthened NCD governance through establishment of NCD unit in the MOH<br>-Strengthened NCD governance through stakeholder engagement of technical working partners and catalyzing partnerships<br>-Reviewed and informed innovative service delivery models for NCDIs
Kenya	-Broadened national NCDI policy through contribution of prioritized interventions to the Kenya Essential Health Package<br>-Built a national coalition through dissemination of findings in a National Stakeholders Meeting (2018)<br>-Informed development of the Kenya NCD Strategic Plan<br>-Participated in global advocacy and network building at World Health Assembly (2018), UN General Assembly (2018), and Prince Mahidol Award Ceremony (2019)
Liberia	-Strengthened NCDI governance through financial and technical support to the NCD Division of the MOH<br>-Built a national coalition through serial convenings and launch event on World Diabetes Day co-hosted with the World Health Organization (Nov 2018)<br>-Supported the planning and scale-up of an integrated NCD service packages
Malawi	-Strengthened NCDI governance through technical support to the MOH NCD Division<br>-Built a national coalition through convening of technical partners and national stakeholders launch meeting (2018)<br>-Diversified national STEPwise survey to include additional conditions (epilepsy, mental health) and a detailed socioeconomic index<br>-Awarded the United National Interagency Task Force Award for excellence in NCD response (2019)<br>-Informed scale up of integrated NCD clinic service models<br>-Contributed to Malawi NCD strategic planning and monitoring frameworks
Mozambique	-Built a national coalition through convening of multi-sectoral stakeholders for national level dissemination meeting (2018)<br>-Developed NCD research collaborations for Mozambique Institute for Health Education and Research with partner organizations

	-Catalyzed regional coalition building through hosting of the *Southern African Regional NCDI Dialogue on Science, Implementation, and Policy* (2018)<br>-Participated in global advocacy and network building at World Health Assembly (2018 & 2019), UN General Assembly (2018)
Nepal	-Built a national coalition through convening of national and provincial level stakeholder meetings (2018 & 2019)<br>-Broadened national NCDI policy through contribution of prioritized NCDI interventions to inform the National Health Insurance benefits package<br>-Facilitated research collaborations through Kathmandu University and Tribuvhan University<br>-Conducted facility-based assessments for integrated NCDI service delivery of prioritized interventions<br>-Participated in global advocacy and network building at World Health Assembly (2018 & 2019), UN Global Assembly (2018) and Prince Mahidol Award Ceremony (2019)
Rwanda	-Supported national and regional coalition building through convening of East African commissions (2018)<br>-Reviewed existing policies and literature in scale of national model for integrated NCD service delivery<br>-Participated in global advocacy and network building at UN General Assembly (2018) and World Health Assembly (2019)
Tanzania	-Built national coalition through stakeholder convening and dissemination of findings (2018, & 2019)<br>-Participation in global advocacy and network building at UN General Assembly (2018)

Supplement to: Gupta N, Mocumbi A, Arwal SH, et al. Priority-setting of health-sector interventions for noncommunicable diseases and injuries to achieve universal health coverage low- and lower-middle income countries. *Glob Health Sci Pract*. 2021;9(3). https://doi.org/10.9745/GHSP-D-21-00035

Supplement Table 4. Selected Quotes from Semi-Structured Interviews with National NCDI Poverty Commissions (Round 1 Commissions), by Thematic Area and Country

Thematic Area	Direct quote (unless indicated otherwise in brackets)	Country
**Successes - reframing**	"Most of the findings that were interesting was the impact on young people, and just how much young people are affected by NCDIs in Afghanistan. This was important for the leadership in the MoPH to be very aware of. Before that, the idea was NCDIs just affected old people, but the data shows that young people are also really affected."	Afghanistan
	"Because we now understood how costly these diseases are – for a lot of us, we did not have this perception before, so this was extremely telling."	Mozambique
	"The major findings were the burden of disease and highlighting for the first time the equity aspects of these diseases. Some diseases are several "fold" more prevalent among certain groups as well as the impoverishment impact of the diseases, which were never previously studied [in Nepal]."	Nepal
	"[Our findings] support the hypothesis that there are NCDs related to poverty, infection, environment and that we need to uncover these risks to address NCDs. It's not just the typical '4x4' narrative."	Tanzania
**Successes – awareness and partnership building**	"[The Commission] was the first time sharing these type of findings for NCDs with national partners – previously this type of information did not exist in Afghanistan. It was important to be able to share these findings with other national partners including WHO [World Health Organization]."	Afghanistan
	"From the government all the way down to the community level, [the commission] drew attention to the role of NCDs. NCDs represent 60-70% of our mortality. [The] ministry is taking the findings and burden of NCDs seriously."	Ethiopia
	"As it was the first time [to have a NCDI Poverty Commission], I recognize that the whole commission's work was innovative, as nothing had been done at the national level on NCDs. The commission has been innovative in bringing together a whole range of specialists and trying to make something happen at a policy level."	Haiti
	"The work of the commission was helpful because it established for the first time a dialogue among several stakeholders [….] that are interested in NCDIs of poverty, as well as of NCDs in general. In that way, it was extremely helpful and were able to produce the first national report."	Mozambique
**Successes – planning**	"The Afghanistan NCDI program is one of the youngest in the MoPH [Ministry of Public Health]. Previously we did not have a strategic plan or an implementation plan, so working on the commission allowed the protected time and some funds to identify some national priorities for beating NCDs"	Afghanistan
	"For the first time, it [the commission] was creating an evidence-based NCDI agenda and will inform a national strategy based on national data."	Ethiopia
	"We were able to show how important it was to introduce NCDs into the health system and into health planning."	Mozambique
	"Traditionally, there is a lack of evidence-based decision-making. Most of the planning is done based on previous experience but not looking at the scientific experience. Not everyone is using data, so this was a good experience because people were responding to data."	Tanzania
**Successes – Governance**	"[The] commission was a success – now we have a national platform that involves the MOH [Ministry of Health]. We now have a common goal whereby we will be able to advocate for the commission recommendations that will benefit the entire nation. […] We see now we don't need to be replicating efforts, but by working together, we were able to have greater outputs."	Malawi
	"I believe that the first year was a good success. We followed up so much that we now have a NCD section in the country now. [We] didn't have a separate NCD section in Nepal previously."	Nepal
**Successes – Civil Society**	"The patient advocacy videos were a great success and have been well received when shared with partners. [It] was important to include the perspective of people from rural areas when looking at national planning."	Ethiopia
	"Using the voice of the people really helps to bring the point across, and it was great to see how interventions were affecting people. This can't be seen by a paper or by conducted research, so you really need to hear that from the people who are living with these conditions."	Tanzania
**Successes – Capacity Building**	"[I] would be interested in continuing to learn from other countries and hoping to share some of the things we have learned, but especially to learn from others who have been doing this for longer and working all over the world."	Afghanistan
	"I would like the Malawi Commission to be a learning hub in the region where other commissions and countries come to learn."	Malawi
	"We have been fairly successful in getting some opportunities through this commission team […]. The secretariat [provided a] learning opportunity for new countries, […] connecting with academic institutions and being a part of this network."	Nepal
	"We'd be happy to build Nepal as a regional hub as commissions for other countries […] and also build capacity of these other countries in a joint way. [We] have a strong presence in academia here."	Nepal
**Facilitating Factors**	"A core coordinator to move things along was extremely helpful."	Ethiopia
	"[A coordinator] really helped the process [and] the management of the Ministry of Health with the NCD team taking some ownership, but the ministry as a whole was very involved with the Minister chairing many of these meetings."	Liberia

Supplement to: Gupta N, Mocumbi A, Arwal SH, et al. Priority-setting of health-sector interventions for noncommunicable diseases and injuries to achieve universal health coverage low- and lower-middle income countries. *Glob Health Sci Pract*. 2021;9(3). https://doi.org/10.9745/GHSP-D-21-00035

Thematic Area	Direct quote (unless indicated otherwise in brackets)	Country
	"Having key government decision makers, involving multiple sectors, and having at least one-key full time staff, and leadership from visionary people is what got the commission started"	Nepal
**Challenges – MOH Turnover / Accountability**	"MSPP [Ministère de la Santé Publique et de la Population] should really be leading this work - we shouldn't be doing it for them. It's so important that they get involved and that they oversee and conduct the programs. Change in leadership within the MSPP presented some significant challenges, particularly as the report had just been finalized as the leadership change happened."	Haiti
	"The ministry are the ones doing the planning at the central level of the planning. We [the commission] are just using our own work to influence policy, rather than shape or make decision"	Mozambique
	"The changes in the government officials was also a challenge, but [the] commission did its best to manage that."	Nepal
	"Initially, the commission was going well, but once there was a change in leadership at MOH [Ministry of Health], it was difficult to bring everyone together"	Tanzania
**Challenges – Commission Resources / Attendance**	"[A] key challenge was that the commissioners were doing voluntary work and that they were not paid."	Ethiopia
	"Regarding the participation of the commission meetings, [it] was difficult to really get everyone together and have final sign off from [everyone] every time."	Haiti
	"The commissioners were all committed to other engagements, [so] time was an issue for them. We had 28 commissioners, but there were only a few that were there for every meeting."	Malawi
	"I think we could be more active and more successful if we had full support of our own leadership at respective institutions."	Mozambique
	"One of the challenges is making all of the commissioners active but then recognizing that it's not possible to engage everyone. We are asking people to engage above and beyond their commitments."	Nepal
**Challenges – How to move to action?**	"It is unclear if the way that the findings are being applied will be successful. Even if we plan, with so much political uncertainty, it's unclear what will happen, but we have no choice but to move this forward."	Haiti
	"How to prioritize the work has been a challenge. We will not be able to do so much and that can be frustrating."	Liberia
	"Currently, at the central government, there is no budget line for NCDs. We need to have a collaborative effort to make sure these recommendations are put into action. Based on the findings from the Global Burden of Disease, we need more investment."	Malawi
	"We have made a mark with policymakers, but how do we reach people living in the community. This is where we need to go next."	Malawi
	"One of the other challenges was thinking about what happens after the report? What is next? How do we implement the findings and build them into policy?"	Malawi
